# Wound Healing in Human Skin Equivalents Reconstructed with Biopolymers Under Fine-Dust Exposure

**DOI:** 10.3390/polym17070901

**Published:** 2025-03-27

**Authors:** Taeeun Kim, Junwoo Lim, Jaehyun Jeong, Heewook Ryu

**Affiliations:** Department of Chemical Engineering, Soongsil University, 369, Sangdo-Ro, Ronjak-Gu, Seoul 06978, Republic of Korea; taeat@soongsil.ac.kr (T.K.);

**Keywords:** biopolymers, human skin equivalents, fine dust, wound healing

## Abstract

Airborne fine-dust pollution poses a significant threat to both respiratory and skin health; however, the skin’s wound-healing process in response to such exposure remains underexplored. Therefore, this study examined the effect of fine-dust-model compounds, specifically polycyclic aromatic hydrocarbons (PM_10_-PAHs) and trace-metal-containing particles (PM_10_-Trace), on the wound-healing process using human skin equivalents reconstructed with collagen-based biomaterials and human skin cells. Our findings revealed that fine-dust exposure significantly delayed wound closure by 2–3 times compared with unexposed controls, impairing re-epithelialization. Live imaging of wound-healing dynamics revealed that trace-metal-containing particles had a more pronounced inhibitory effect than polycyclic aromatic hydrocarbons. Furthermore, fine-dust exposure elevated protease-activated receptor-1 (PAR1) expression by up to 161%, indicating significant physiological disruption. Additionally, fine-dust exposure triggered inflammation and oxidative stress, leading to structural and functional damage in the reconstructed skin. These results provide critical insights into how airborne pollutants disrupt skin repair mechanisms and highlight the need for targeted strategies to mitigate their harmful effects.

## 1. Introduction

Airborne fine-dust pollution has emerged as a significant environmental and health concern, with its levels rising at an alarming rate [1]. Fine-dust particles, suspended in the atmosphere, not only affect respiratory health but also come into direct contact with the skin, positioning it as one of the primary organs exposed to these pollutants [2]. The structural and physiological integrity of the skin is maintained by epidermal cells and dermal fibroblasts, which are essential for skin development, homeostasis, and repair [3]. Previous studies from our group have investigated the effects of fine-dust exposure on dermal and epidermal cells, highlighting its impact on their physiological responses, including inflammatory reactions and aging processes [4]. However, its effects on wound healing remain largely unexplored.

One of the skin’s critical functions is to serve as a protective barrier against environmental challenges and physical damage, thereby safeguarding the body’s internal environment [5,6]. When the skin is wounded, a series of intricate processes are activated to repair the damage and restore its original state. These processes are essential for maintaining the skin’s integrity and barrier functions, which are vital for preventing microbial invasion, retaining body fluids, and supporting immune defense [7,8]. However, impaired wound healing can lead to bacterial proliferation, further delaying recovery and potentially causing chronic wounds characterized by sustained inflammation, hindered keratinocyte migration, and delayed re-epithelialization [9,10].

Fine dust, an environmental pollutant composed of particles smaller than 10 μm in diameter, has been associated with various health issues, including respiratory diseases, cardiovascular conditions, inflammation, oxidative stress, and neurodegenerative disorders [11,12,13]. While much attention has been given to its impact on systemic health, the direct effects of fine dust on wound healing remain largely underexplored. Emerging evidence suggests that fine-dust exposure induces the production of inflammatory mediators such as cyclooxygenase-2, prostaglandin E2, interleukin (IL)-1β, and IL-6 in keratinocytes, which disrupts skin barrier function, promotes oxidative stress, and accelerates atopic dermatitis progression [14]. Additionally, fine-dust exposure stimulates the expression of matrix metalloproteinase-1 (MMP-1), which degrades collagen and accelerates premature skin aging [15]. Given that inflammation and oxidative stress are key regulators of wound healing, these findings suggest that fine dust may directly impair the re-epithelialization process, delay wound closure, and influence the expression of wound-healing markers, potentially increasing the risk of chronic wound formation.

Traditional animal models and in vivo human trials present significant challenges in evaluating the effects of environmental pollutants like fine dust on skin wound healing. Animal models often fail to accurately replicate human-specific responses due to species differences in skin structure, immune reactions, and repair mechanisms. Additionally, ethical concerns and regulatory restrictions have led to a decline in the use of animal models for dermatological and toxicological research. While in vivo human trials offer clinical relevance, they pose ethical and practical limitations, particularly when studying airborne pollutants that may pose health risks to participants. These challenges underscore the need for a reproducible, physiologically relevant, and ethically viable alternative.

**Figure 1:**
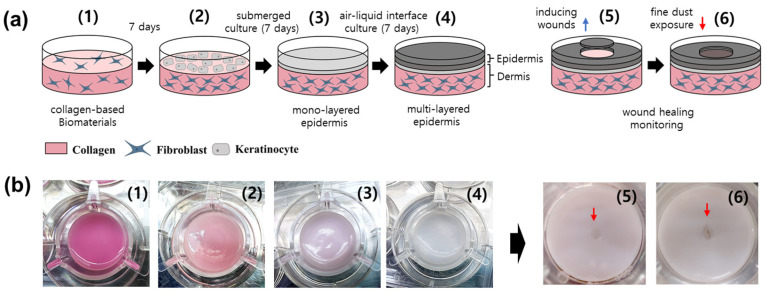
(**a**) The entire process scheme of reconstructing the human skin equivalent and its application for monitoring wound healing under fine-dust exposure. Initially, the collagen solution was mixed with the dermal cell suspension to prepare the dermal layer, followed by a 7-day incubation period (1). Keratinocytes were then cultured on the dermal layer (2) until a monolayer was formed (3), and subsequently exposed to air to promote differentiation (4). The full-thickness skin equivalent was reconstructed, after which wounds were created using a biopsy punch (5), and fine dust was applied to monitor the wound-healing process (6). (**b**) The actual manufacturing steps of each stage in reconstructing the human skin equivalent and the wound-healing monitoring process are illustrated. Arrows shown in steps (5) and (6) indicate the wound sites.

This study aimed to examine the effect of fine-dust-model compounds, specifically PM_10_-PAHs and PM_10_-Trace, on the wound-healing process using human skin equivalents reconstructed with collagen-based biopolymers and human skin cells (Figure 1). Several commercially available reconstructed human skin equivalents are used in clinical treatments and in vitro testing. Models such as Apligraf^®^, Epicel^®^, Dermagraft^®^, and Integra^®^ are employed for wound healing and burn treatments, while EpiDerm™, SkinEthic™ RHE, and EpiSkin™ are widely utilized in cosmetics and pharmaceutical research for skin irritation and penetration assessments [16,17,18]. However, despite their extensive use, studies examining the reconstructed human skin equivalents’ responses to airborne fine-dust exposure remain limited. This study aimed to bridge this gap by evaluating the wound-healing process of human skin exposed to fine dust pollutants, offering new insights into the impact of environmental factors on skin repair mechanisms. To address this gap, we investigated the impact of fine dust on wound healing using full-thickness human skin equivalents, which closely mimic the structure and function of human skin. Typically reconstructed with keratinocytes cultured on a dermal substitute at the air–liquid interface, these models provide an effective platform for assessing skin recovery dynamics. By utilizing these reconstructed human skin equivalents, we monitored physical recovery patterns, such as wound closure area and healing speed, and evaluated physiological responses, including protease-activated receptor-1 (PAR1) expression, during the wound-healing process. These findings are expected to contribute to a deeper understanding of human skin’s wound healing mechanisms and provide a foundation for assessing the effects of environmental pollutants like fine dust on wound recovery using ethical and effective in vitro models.

## 2. Experiments

### 2.1. Cell Culture of Human Skin Cells

To construct reconstructed human skin equivalents, fibroblasts and keratinocytes were cultured. Fibroblasts (HDFn, neonatal, Gibco, Grand Island, NY, USA) were maintained in a 75 cm^2^ flask with growth medium (M106, Thermo Fisher Scientific, Waltham, MA, USA) supplemented with 10% Low-Serum Growth Supplement (LSGS, Gibco, NY, USA) and 1% penicillin–streptomycin (P/S, 100×, Biowest, Paris, France). Keratinocytes (HEKn, neonatal, Gibco, NY, USA) were cultured in EpiLife™ medium (60 µM calcium, Gibco, NY, USA) supplemented with Human Keratinocyte Growth Supplement (HKGS, Gibco, NY, USA) and 1% P/S. Both cell types were incubated at 37 °C, 5% CO_2_, with media changes every two days. Upon reaching 80% confluence, cells were detached using 0.25% trypsin (Biowest, France) and used for experiments. Cell morphology was observed under an optical microscope (Nikon Eclipse TS100, Tokyo, Japan).

### 2.2. Human Skin Equivalents Reconstructed with Collagen-Based Biomaterials

Reconstructed human skin equivalents consisted of a fibroblast-seeded collagen matrix forming the dermal layer and a keratinocyte layer differentiated into the epidermis. The dermal matrix solution was prepared by mixing HDFn cells with collagen type I (6.0 mg/mL, Advanced Bio-Matrix, Carlsbad, CA, USA), Dulbecco’s modified Eagle medium (DMEM, Gibco), Ham’s F-12, NaHCO_3_, and NaOH. The mixture was cast in transwells (0.4 μm pores) and gelled for 2 h at 37 °C, 5% CO_2_. The dermis was submerged in fibroblast growth medium (M106) for 6 days with media changes every two days. Keratinocytes (HEKn) were seeded on the dermal surface and submerged in EpiLife™ medium for 3 days to form a monolayer. Further differentiation was induced using CnT-PR-FTAL5 medium (CELLnTEC, Bern, Switzerland) for 1 day, followed by air–liquid interface culture to establish the cornified epidermis. The integrity of the skin equivalents was assessed through transepithelial/endothelial electrical resistance (TEER) measurements using an EVOM3 Epithelial Volt/Ohm Meter (World Precision Instruments, Sarasota, FL, USA). Sterilized electrodes and a custom holder were used to ensure accurate measurements, performed by filling the transwells with PBS and subtracting the resistance of PBS-only controls. Measurements were conducted in triplicate to ensure reliability. To ensure a sterile environment, all reconstructed human skin equivalents were maintained in penicillin–streptomycin (PS)-supplemented culture medium, and all experiments were conducted under clean bench conditions and incubators. Additionally, fine dust samples were UV-sterilized prior to exposure to eliminate potential microbial contamination.

### 2.3. Wound-Healing Model and Analysis of Particulate-Matter Exposure

A wound-healing model was developed by introducing wounds (3 mm diameter) into the cornified layer of differentiated keratinocytes using a sterile biopsy punch. The wound states were monitored under an optical microscope (Nikon Eclipse TS100, Japan) and a stereo microscope (Olympus SZ61, Tokyo, Japan). To evaluate the effects of particulate matter (PM), PM_10_-PAHs (ERMCZ100, Sigma-Aldrich, St. Louis, MO, USA) and PM_10_-Trace (ERMCZ120, Sigma-Aldrich, MO, USA) were used. PM was sterilized with UV for 30 min, prepared as a 500 μg/mL stock solution in PBS, and dispersed via sonication for 1 h. Final concentrations of 20 and 400 μg/mL were applied to the wounds. Wound closure was monitored every 6 h until complete healing, and morphological analysis was conducted using ImageJ software (version 1.54m) to quantify the wound area and healing rate. Hematoxylin and eosin (H&E) staining was performed on formalin-fixed sections at 0 and 48 h post-wounding to assess structural changes.

### 2.4. Physiological Activity of Wound Healing

The role of protease-activated receptor-1 (PAR1) in wound healing was analyzed using a Human PAR1 ELISA Kit (ab283544, Abcam, Cambridge, MA, USA). Skin equivalents exposed to PM10-PAHs and PM10-Trace (20, 400 μg/mL) were cultured, and culture supernatants were collected at 12, 24, 48, 72, and 96 h, then stored at −40 °C. The ELISA was performed following the manufacturer’s protocol, and absorbance at 450 nm was measured using a microplate reader (Thermo Scientific, Waltham, MA, USA). PAR1 expression levels were quantified using a standard curve to assess the physiological effects of PM exposure on wound healing.

### 2.5. Statistical Analysis

Statistical significance was determined using one-way ANOVA followed by Tukey’s multiple comparison test (* *p* < 0.1, ** *p* < 0.05, *** *p* < 0.01).

## 3. Results and Discussion

### 3.1. Validation of Integrity and Wound Healing in Reconstructed Human Skin Equivalents

**Figure 2:**
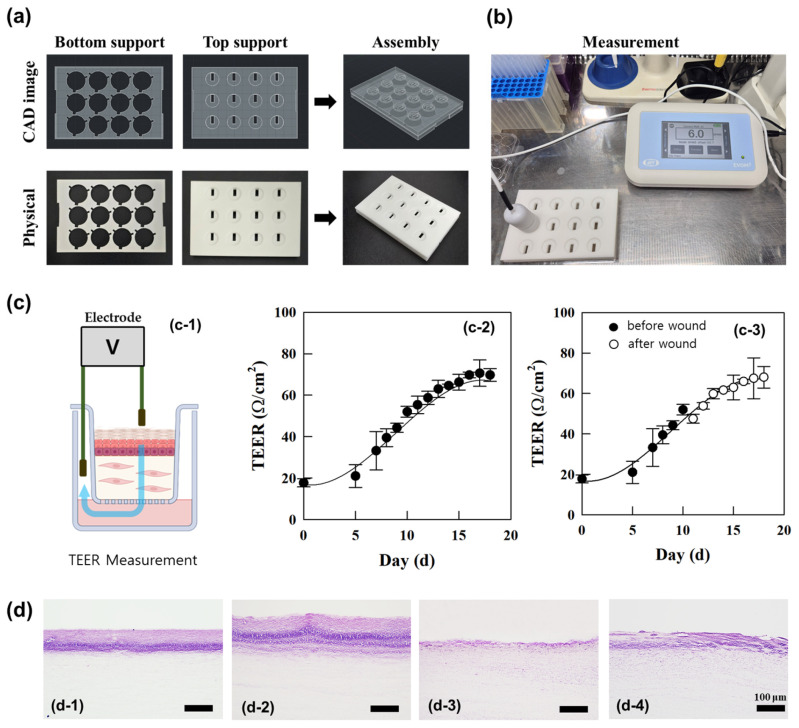
(**a**) Transepithelial electrical resistance (TEER) measurements of full-thickness human skin equivalents were taken after fabricating a support for fixing to a plate with transwells. (**b**) Fixing the plate and wells with the support for stable TEER measurement. (**c**) The integrity of reconstructed human skin equivalents (**c-2**) and the wound-healing process (**c-3**) was assessed by measuring TEER (**c-1**). (**d**) Histological cross-sections of reconstructed human skin equivalents and the wound-healing process are shown: (**d-1**) represents the epidermal formation of reconstructed human skin equivalents; (**d-2**) depicts the fully stratified epidermis with keratinized layers in the final reconstructed skin equivalents; (**d-3**) shows the full-thickness skin equivalent after wound creation using a biopsy punch; and (**d-4**) illustrates the wound-healing process, visualized by H&E staining. The scale bar in (**d-1**) to (**d-4**) represents 100 µm.

**Figure 3:**
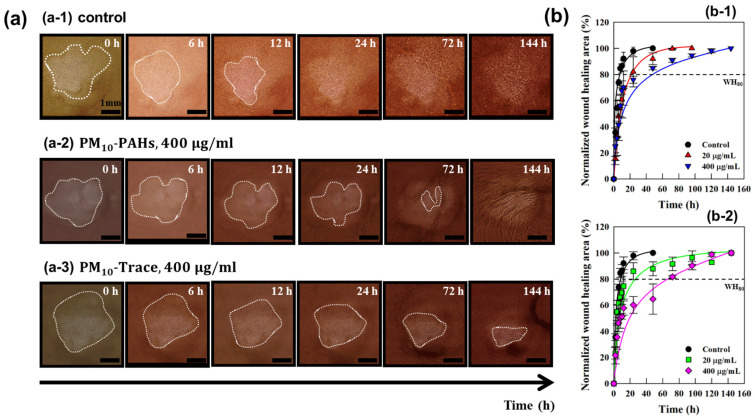
(**a**) The wound-healing process of full-thickness human skin equivalents is shown over 144 h, the maximum healing time point, under exposure to (**a-1**) control conditions, (**a-2**) PM_10_-PAHs, and (**a-3**) PM_10_-Trace at a concentration of 400 μg/mL each. (**b**) Changes in the wound healing area of full-thickness human skin equivalents exposed to (**b-1**) PM_10_-PAHs and (**b-2**) PM_10_-Trace at concentrations of 20 μg/mL and 400 μg/mL are presented from the initial wound treatment to complete healing.

**Figure 4:**
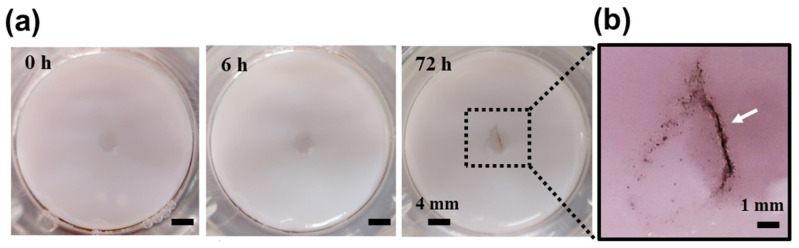
(**a**) The wound-healing process of full-thickness human skin equivalents exposed to 400 μg/mL of PM10-Trace, a component of PMs, is visualized under a stereo microscope. As the wound healed toward the center, PMs are shown adhering to the wound edges and accumulating at the center. (**b**) Enlarged images highlight the PMs gathered at the wound edges and within the wound area. The arrows in (**b**) indicate the accumulation of PMs at the wound edges and center.

In this study, a full-thickness cultured skin model mimicking human skin was developed to investigate wound-healing dynamics [Figure 1]. A 3 mm diameter epidermal wound was induced using a biopsy punch, ensuring consistency in wound healing analysis by excluding samples with dermal damage. Histological evaluations were performed on samples immediately post-wounding and 48 h thereafter using hematoxylin and eosin (H&E) staining alongside control models [Figure 2(d-2)]. The control model, without induced wounds, exhibited keratinized differentiation of the stratum corneum after 48 h [Figure 2(d-4)]. This indicated the successful stepwise reproduction of keratinization and differentiation processes in the epidermis, akin to human skin. Immediately after wound induction, the epidermis at the wound site was removed, leaving the underlying dermis exposed. After 48 h, significant re-epithelialization of the wound surface was observed, emphasizing the structural and physiological integrity of the cultured model. This re-epithelialization aligned with known wound healing mechanisms, where keratinocyte migration, differentiation, and keratinization are critical [19]. Keratinocytes play a pivotal role in covering wound surfaces and promoting healing by migrating and differentiating [20].

The structural integrity and wound healing capacity of the full-thickness cultured skin model were assessed through TEER (transepithelial electrical resistance) measurements, which quantitatively evaluated barrier function. Prior to measurement, a support structure was fabricated and used to fix the plate and transwells for stable TEER assessment, ensuring consistent and reliable resistance readings [Figure 2a,b]. Higher TEER values correlated with enhanced tissue integrity and wound recovery [21]. TEER was measured using an EVOM3 Epithelial Volt/Ohm Meter (World Precision Instruments, Sarasota, FL, USA) [Figure 2a]. Resistance values were calculated by subtracting blank readings and multiplying by the transwell area. As keratinocytes proliferated and differentiated on the collagen support layer, gradual increases in resistance were observed. By day 8, at the air–liquid interface, TEER values showed a 123% increase compared with day 0 [Figure 2(c-2)]. This trend underscored the contribution of epidermal layer formation to the increase in TEER values. On day 11, wounds were induced in the epidermal layer of the cultured skin, and the TEER values of the wounded model were compared with the unwounded control [Figure 2(c-3)]. Immediately post-wounding, TEER values in the wounded model were 14% lower than the control. By day 12, resistance values increased by 13% compared with immediately post-wounding. By day 18, TEER values of the wounded model reached 68 Ω/cm^2^, closely aligning with the control value of 70 Ω/cm^2^, representing a 43% increase from the immediate post-wounding value of 47 Ω/cm^2^. These findings indicate that the epidermal barrier in the full-thickness cultured skin wound model substantially recovered. The increase in TEER values during wound healing reflected epidermal barrier formation and recovery [22]. The correlation between TEER values and re-epithelialization highlights TEER’s potential as a non-invasive tool for monitoring wound healing [23]. In summary, the observed increase in TEER values during keratinocyte differentiation, epidermal formation, and wound healing suggests its utility as a quantitative, non-invasive indicator for evaluating the integrity and recovery of full-thickness cultured skin models.

These findings reinforce the validity of reconstructed human skin equivalents as a reliable model for studying wound-healing dynamics. The observed TEER recovery trend, alongside histological evidence of re-epithelialization, confirms that the cultured skin model effectively mimicked epidermal barrier restoration following injury. Furthermore, the quantitative nature of TEER measurements provided a non-invasive and reproducible approach for evaluating wound healing efficiency in vitro. Future studies could explore the integration of additional wound healing biomarkers, such as inflammatory cytokines or extracellular matrix remodeling proteins, to further elucidate the molecular mechanisms involved in fine-dust-induced wound healing impairment.

### 3.2. Wound Healing in Human Skin Equivalents Exposed to Fine Dust

This study assessed the impact of fine dust (PM_10_-PAHs, PM_10_-Trace) on wound healing using a 3 mm epidermal wound in full-thickness cultured skin. Fine dust components were applied at concentrations of 20 μg/mL and 400 μg/mL, and wound healing was monitored over 144 h using optical and stereoscopic microscopy [Figure 3a]. In the control group (no fine-dust exposure), wound closure occurred within 12 h, with 90% of the wound area healed. Conversely, samples exposed to 400 μg/mL of PM_10_-PAHs and PM_10_-Trace exhibited delayed wound healing, requiring approximately 90 h to achieve similar closure, a 650% increase in healing time compared with the control [Figure 3b]. These results indicate the detrimental effects of fine-dust exposure on wound healing in full-thickness cultured skin. Previous studies have reported re-epithelialization in mammalian skin wounds via keratinocyte proliferation and migration from wound edges to the center [24,25]. Similar patterns were observed in the current study, with wound closure originating from the edges in both control and fine-dust-exposed samples.

Fine-dust particles adhered to the wound edges and migrated toward the wound center during healing [Figure 4]. This supports the critical role of keratinocyte migration and proliferation in wound recovery and suggests that the cultured skin model replicated in vivo wound healing patterns. Fine-dust exposure negatively impacted keratinocyte proliferation and migration, delaying wound healing. PAHs, a major environmental pollutant, are known to induce oxidative stress and inflammatory responses, impairing cellular functions [4]. Fine-dust exposure may indirectly influence cortisol secretion by activating the hypothalamic−pituitary−adrenal (HPA) axis, further inhibiting keratinocyte migration and prolonging wound recovery [26]. Heavy metals in fine dust exacerbate cellular toxicity, disrupting normal keratinocyte function and delaying wound healing [27]. These findings emphasize the potential risks of fine-dust exposure to skin health.

These results highlight the significant impairment of wound healing caused by fine-dust exposure, underscoring the potential health risks posed by environmental pollutants. The observed delay in wound closure and disruption of keratinocyte function suggest that fine-dust exposure compromises the skin’s regenerative capacity, potentially increasing the risk of chronic wounds or impaired barrier recovery. Given the widespread presence of fine dust in urban environments, further investigations into the molecular mechanisms underlying these effects, including the role of oxidative stress, inflammatory pathways, and cellular senescence, are essential for developing targeted therapeutic interventions to mitigate the impact of air pollutants on skin health.

### 3.3. Assessment of Wound Recovery in Fine-Dust-Exposed Human Skin Equivalents

**Figure 5:**
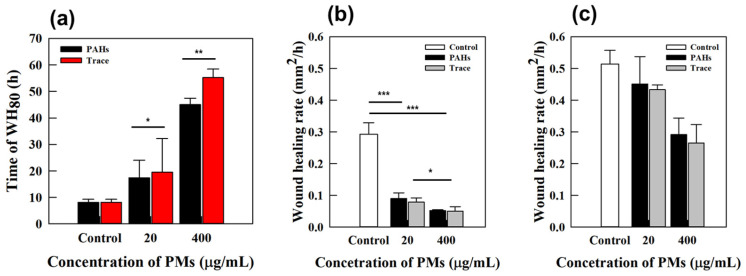
(**a**) The time to 80% wound-area healing for a full-thickness human skin equivalent is shown. (**b**) The rate of recovery during the first 12 h and (**c**) the time to full recovery are presented as the healing area per hour after wounding, with full-thickness human skin equivalents exposed to PM10-PAHs and PM10-Trace at concentrations of 20 and 400 μg/mL for each PMs component. (* *p* < 0.1, ** *p* < 0.05, *** *p* < 0.01).

Using full-thickness cultured skin, the effects of fine dust (PM_10_-PAHs, PM_10_-Trace) on wound healing were evaluated. Fine dust components were applied at 20 μg/mL and 400 μg/mL, and wound recovery was monitored for up to 144 h using optical microscopy. Wound areas were measured at different time points using ImageJ software, and recovery rates and durations were quantitatively analyzed [Figure 5]. Control samples demonstrated rapid wound closure within 48 h, achieving 100% recovery. In contrast, fine-dust-exposed samples required 96 to 144 h for complete healing, representing a 2- to 3-fold delay compared with controls. This underscores the impact of fine dust on wound healing duration. Localized factors, including oxidative stress and inflammation, likely impair keratinocyte proliferation and migration, contributing to delayed recovery. Fine dust components, particularly PAHs and heavy metals, further disrupt cellular processes, emphasizing the health risks posed by environmental pollutants.

The prolonged wound healing observed in fine-dust-exposed samples highlights the detrimental impact of environmental pollutants on skin regeneration and barrier restoration. The delayed recovery suggests that persistent oxidative stress and inflammation induced by fine dust may compromise cellular repair mechanisms, potentially leading to chronic wound conditions. Future studies should investigate targeted strategies to counteract fine-dust-induced skin damage, including antioxidant treatments or barrier-enhancing formulations, to mitigate the adverse effects of airborne pollutants on wound healing.

### 3.4. Physiological Validation of Wound Healing in Full-Thickness Cultured Skin

**Figure 6:**
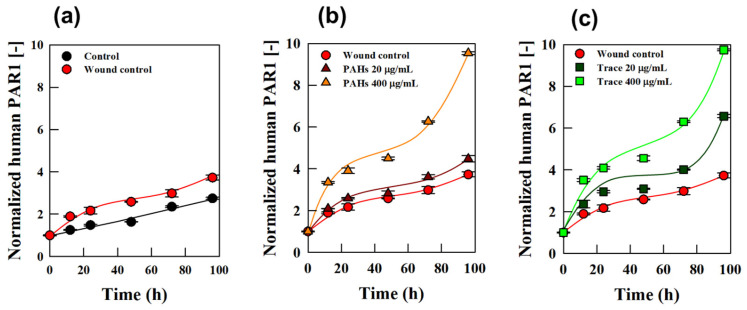
The analysis of PAR1 expression in full-thickness human skin equivalents and wound models. (**a**) Compares PAR1 expression levels between unwounded controls and wound models to highlight PAR1’s role during the wound-healing process. (**b**) Shows the impact of PM_10_-PAHs on PAR1 expression in wound models at various concentrations. (**c**) Presents the effects of PM_10_-Trace on PAR1 expression, demonstrating its differential influence compared with PM_10_-PAHs.

The stability of the cultured skin was assessed by evaluating its structural integrity, confirming that it remained stable for over 6 weeks after fine-dust exposure. Given this long-term stability, the wound healing experiment was conducted for 2 weeks post-exposure to specifically assess the effects of fine dust on skin repair. During this period, we analyzed the rate of re-epithelialization and the expression of key wound-healing markers, including protease-activated receptor-1 (PAR1), to evaluate how fine dust influenced the wound-healing process. This study analyzed the expression of protease-activated receptor-1 (PAR1) during the wound-healing process and its modulation by fine-dust exposure. PAR1, in conjunction with the endothelial protein C receptor (EPCR), mimics the effects of activated protein C (APC). This interaction exerts anti-inflammatory effects on endothelial cells and strengthens barrier functions, playing a critical role in wound healing. Additionally, PAR1 mediates keratinocyte proliferation, facilitating tissue repair and accelerating wound recovery [28]. To assess PAR1 expression, culture media from full-thickness skin models were collected at 12, 24, 48, 72, and 96 h post-wounding, and PAR1 levels were analyzed [Figure 6]. When comparing control and wound models, PAR1 expression in the wound model increased by 51% at 12 h and by 58% at 48 h [Figure 6a], indicating an upregulation of PAR1 during wound healing. This aligns with its role in promoting cell migration and proliferation during recovery [29], highlighting the feasibility of using full-thickness cultured skin for evaluating wound healing.

The effects of fine dust on wound healing were further evaluated by comparing PAR1 expression in wound models exposed to PM_10_-PAHs and PM_10_-Trace with non-exposed models [Figure 6b,c]. Fine-dust exposure significantly increased PAR1 expression, particularly at a high concentration of 400 μg/mL. At 12 h post-exposure, PAR1 expression increased by 77% with PM_10_-PAHs and by 86% with PM_10_-Trace. By 96 h, PM_10_-PAHs resulted in a 156% increase, while PM_10_-Trace caused a 161% increase compared with the control. These findings suggest that fine-dust exposure enhances PAR1 expression, indicating its impact on the wound-healing process. Interestingly, wounds exposed to PM_10_-Trace exhibited higher PAR1 expression levels than those exposed to PM_10_-PAHs. For example, at 96 h, wounds treated with 20 μg/mL of PM_10_-Trace showed a 47% greater increase in PAR1 expression compared with PM_10_-PAHs at the same concentration. This observation is consistent with the trend in wound recovery rates presented in Figure 5, highlighting the differential effects of fine dust components on healing dynamics. These results suggest that PM_10_-Trace may exert more pronounced physiological effects on wound healing compared with PM_10_-PAHs. Fine dust appears to disrupt the wound-healing process by increasing inflammation and interfering with physiological pathways critical to recovery [30]. This underscores the complex interplay between cellular mechanisms and environmental pollutants [31], emphasizing the need for further investigation into the health impacts of fine-dust exposure.

Reconstructed human skin equivalents play a significant role in clinical treatments, pharmaceutical testing, and cosmetic industries as ethical and cost-effective alternatives to animal models. The use of reconstructed human skin equivalents as an in vitro model provides a human-relevant, ethical, and reproducible platform for evaluating the biological effects of environmental pollutants. Unlike traditional in vivo animal models, which often suffer from species differences, ethical concerns, and variability in exposure conditions, the cultured skin models enable precise mechanistic studies in a controlled environment. The structural and physiological differences between rodent and human skin also limit the direct translatability of animal-model findings to human skin health. By expanding their application to assess wound healing under fine-dust exposure, this study highlights a previously underexplored aspect of environmental toxicology [32]. Given the increasing concerns over airborne pollution and its impact on skin health, our findings suggest that the reconstructed human skin equivalents could be further utilized for evaluating environmental stressors, potentially broadening their market relevance in toxicology and dermatological research.

## 4. Conclusions

This study demonstrated that fine-dust exposure significantly delayed wound healing in full-thickness human skin equivalents, reconstructed to mimic the structure and function of human skin. The engineered skin models were validated for their structural integrity and suitability as a wound-healing platform using H&E staining, TEER measurements, and PAR1 expression analysis. Fine-dust exposure, particularly to PM_10_-PAHs and PM_10_-Trace, prolonged wound closure times by 2–3 times compared with unexposed controls, with recovery delayed by 1.1-fold and 1.3-fold at low and high concentrations, respectively. Elevated PAR1 expression by up to 161% in high-concentration exposures further highlighted the physiological disruptions caused by fine dust. These findings reveal that fine dust impairs the skin’s repair mechanisms, not only by delaying re-epithelialization but also by inducing structural and functional damage. This study also emphasizes the potential of full-thickness skin equivalents as a reproducible and ethical alternative to animal testing for investigating the impact of environmental pollutants on skin health.

## Data Availability

Data are contained within the article.
